# Cellular precision modulation of JAK1: tailoring therapies for allergic lung inflammation

**DOI:** 10.1038/s41392-024-01843-y

**Published:** 2024-06-17

**Authors:** Xiaocheng Yan, Siegfried Ussar

**Affiliations:** 1 Research Unit Adipocytes & Metabolism (ADM), Helmholtz Diabetes Center, Helmholtz Zentrum München, German Research Center for Environmental Health GmbH, 85764 Neuherberg, Germany; 2 German Center for Diabetes Research (DZD), 85764 Neuherberg, Germany

**Keywords:** Immunological disorders, Immunological disorders

In a recent publication published in *Cell*, Tamari et al. unraveled a previously unrecognized role of JAK1 signaling in vagal sensory neurons regulating the immune response to allergic lung inflammation.^1^ The authors show that sensory neuromodulation could be an important therapeutic avenue for the treatment of lung inflammation.

It is now well established that the number of patients suffering from allergic disorders, like those suffering from obesity and the metabolic syndrome, is rapidly increasing world-wide, accelerating the burden on individual health and healthcare systems. Thus, basic and clinical researchers urgently need to develop novel therapeutics and approaches to limit or prevent disease development or progression. Among those, inhibitors of Janus kinase (JAK) represent promising treatment options for various inflammatory and other diseases, including allergic disorders. Due to the diverse functions of JAK family members in various tissues and cell types, JAK1-selective inhibitors were developed and are used for the treatment of atopic dermatitis but remain under clinical evaluation for the treatment of asthma. However, systemic inhibition of Janus kinases in general or JAK1 specifically remains a challenge with regards to unclear side-effects due to functions in off-target tissues and cell types.^2^

To better understand the systemic effects of JAK1 activation, Tamari et al. generated a mouse model replacing murine Jak1 with a human JAK1 gain of function variant (p.A634D) that resulted in spontaneous atopic dermatitis-like inflammation. However, lung inflammation, as seen in patients expressing this variant, was only observed upon aeroallergen exposure, indicating differences between skin and the lung in disease development and progression. In an elegant set of experiments using various transgenic mouse lines, bone marrow chimeras and chemical denervation, Tamari et al. demonstrated that increased JAK1 activity in vagal sensory neurons suppresses allergic lung inflammation. The authors show that JAK1 activation in non-hematopoietic cells, using wildtype bone marrow chimeras, confers resistance to lung inflammation. Conversely, chemical inhibition of sensory neurons arising from the vagal ganglia (VG) strongly exaggerated, lung inflammation induced by the potent lung allergen *Alternaria alternata*. No effects were observed upon impairment of dorsal root ganglion sensory neurons. In line with these findings, intraperitoneal administration of the JAK-1 selective inhibitor upadacitinib resulted in an overall improved lung pathology in *Alternaria alternata*-induced mice, while intranasal administration had strongly reduced effects. These experiments demonstrate that intranasal administration of the JAK-1 inhibitor failed to effectively suppress allergic lung inflammation due to limited drug distribution within the airways and preferential access to sensory neurons, while systemic delivery also targeted hematopoietic cells.

**Fig. 1:**
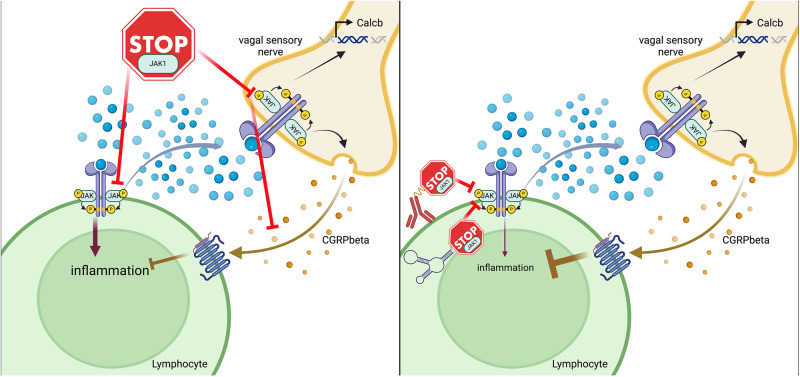
Cell-type-specific inhibition of JAK1 could improve therapeutic efficacy in allergic lung inflammation. **(left side)** Systemic inhibition of JAK1 reduced inflammatory responses in lymphocytes but is compensated by reduced anti-inflammatory actions of vagal sensory nerves in context of allergic lung inflammation. (**right side**) Lymphocyte-restricted JAK1 inhibition through targeted delivery of JAK1 inhibitors via antibodies, aptamers or other molecules could overcome this limitation. Selective inhibition of JAK1 in lymphocytes could inhibit the pro-inflammatory actions in these cells while retaining the anti-inflammatory actions through CGRPβ in vagal sensory nerves. Created with Biorender.com

Mechanistically, the authors find that JAK1 activity in vagal sensory neurons regulates expression and release of the neuropeptide calcitonin gene-related peptide beta (CGRPβ), which suppresses pathogenic group 2 innate lymphoid cell (ILC2) responses. To confirm this, intranasal administration of CGRPβ to *Alternaria alternata* treated Rag1 knockout mice, where allergic lung inflammation depends on ILC2s, attenuated immune cell responses compared to control vehicle-treated Rag1 knockout mice. This further strengthens the authors’ conclusions that CGRPβ secreted from vagal sensory neurons indeed suppresses allergic immune responses. Taken together, Tamari et al. provide convincing evidence that inhibition of JAK1 not only suppresses immune cell activation but also the immune modulatory function of sensory neurons in response to lung allergens (Fig. 1 left side). Similar findings on positive effects of both agonism and antagonism are also seen in completely different fields, such as for the GIP receptor,^3^ which recently gained great interest due to the approval of GLP-1/GIP dual agonist tirzepatide for treatment of type 2 diabetes and body weight loss. These examples highlight the inherent complexity of pharmacologically targeting important signaling pathways systemically, due to pleiotropic and potentially opposing effects in various tissues and cell types.

It is very important to consider the limitations of this study as outlined by Tamari et al., indicating that validation of the described mechanisms in humans is still pending. Moreover, we want to raise attention that the described experiments were conducted in lean adult, but not old mice, and without clear distinction of gender. The importance of this in context of allergic lung inflammation is illustrated by the positive association of insulin resistance with asthma,^4^ which differs between genders and age groups. Obesity could also have an impact on the observed mechanisms, as adipose derived leptin increases with fat mass gain and signals through the leptin receptor via the JAK/STAT pathway. As the leptin receptor is expressed on vagal sensory neurons, obesity associated hyperleptinemia could therefore impact on the mechanism described here or change CGRPβ expression. It will be interesting to see the contribution of vagal sensory neuron JAK1 signaling in mice with co-morbidities, such as obesity, insulin resistance and others, that often co-occur in patients.

Nevertheless, the data provided by Tamari et al., are a good example that systemic delivery of drugs could result in sub maximal therapeutic efficacy due to counter acting, on-target, effects of the drugs. Here, a tempting approach would be to combine administration of JAK1 inhibitors with CGRPβ or CGRP receptor agonists. However, although the data from Tamari et al., would make a strong argument for such a combination therapy, the broad effects of CGRP agonism, especially its role in migraine would require a more targeted delivery. The lung, like the skin, is in principle easy to selectively target through inhalation. However, the potential beneficial or detrimental role of CGRP action in pulmonary diseases remains controversially discussed.^5^ In addition, the combination of two drugs with known non-overlapping side effects could make approval challenging, which further highlights the necessity of generating detailed understanding of the underlying biology. To this end, targeted delivery of JAK1 inhibitors to lymphocytes, or at least excluding delivery to neurons, could provide a very attractive alternative approach, which should greatly enhance therapeutic efficacy for the treatment of allergic lung inflammation (Fig. 1 right side). The identification and development of tissue selective drug delivery vehicles has been very challenging until now. However, it is our strong belief that concentrated efforts to create antibodies, aptamers, peptides, or small molecules delivering cargo to specific cell types or at least groups of cell types will greatly advance drug development for almost all diseases in the future.
